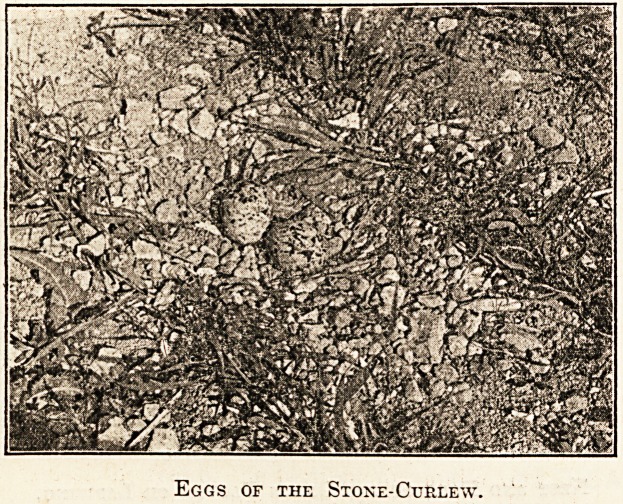# Green Plover and Stone-Curlew

**Published:** 1908-06-06

**Authors:** 


### The Practitioner's Relaxations and Hobbies.

### GREEN PLOVER AND STONE-CURLEW.

The life of the country practitioner has its drawbacks, but it has also its advantages over that of his colleagues in the town. Not the least of these is the fact that he can do much of his work on horseback, for, in the most enclosed country there are usually horse-paths by the road-side and bridle tracks across the fields, and the doctor is seldom reckoned a trespasser even if he forsakes the path.

The country practitioner ought to ride. It is much that he is thus able to secure all the benefits of fresh air and exercise in the performance of his professional duties, but over and above these he may enjoy the pleasure of seeing much that others seldom, or never, see. It is a fact well known, at any rate to those who live in the country, that wild animals of all kinds are, as a rule, much less shy of a man on horseback than of a man on foot, and will, consequently, allow themselves to be approached more nearly and observed more closely by the mounted than by the unmounted traveller. To the man who rides about the country and uses his eyes there will, for this reason, often be revealed, with little effort on his part, facts which the pedestrian only discovers by patient watching, and the man who goes about his work in a carriage or a motor-car not at all. The man who aspires to be a scientific naturalist must, no doubt, do most of his work on foot, but one may take an intelligent interest in the common sights of the country without being a profound naturalist, and, indeed, the country practitioner has not as a rule time to do more than observe in passing that which presents itself to an observant eye.

If our country practitioner is fortunate enough to live in an open down country, he will find in the habits of the common green plovers, variously called lapwings or peewits, a most interesting field of observation. With them the nesting season begins early. Many of them have by the first week of April laid and been robbed of their first clutches of eggs. Others are busy with the dalliance of courtship, which, so far as the male is concerned, consists chiefly of much bowing and turning and scraping of shallow holes in the earth. One of these will eventually be used for nesting purposes, but the majority of them will remain unoccupied, and, having borne testimony for a while to his gallantry, will be obliterated by the rain and the growth of the spring grass. The completed nest is a very poor sort of affair—merely one of these shallow depressions with a few stems of grass and bents placed in it. As to the eggs, they do not need description, for, though perhaps comparatively few of those who read these words will have seen the eggs in the nest, we are all familiar with the outward appearance, at least, of the much coveted delicacies displayed every spring in baskets of moss in the poulterers' shops. The eggs are usually four in number, and are so placed in the nest that their sharp points converge towards the centre. Their colour is such that it blends equally well with surroundings of grass or bare earth, making them, to the inexperienced eye, most difficult to distinguish. Thousands upon thousands of these eggs are to be found in early April by those that have the eyes to see them, and many are there who have such eyes, busy day after day, and all day in the season, hunting for them, for the eggs command a ready market, and the traffic in them in most profitable.

Although the nesting season is at its height in April, fresh eggs may be found even as late as June, for when the plover has been robbed of its first clutch of eggs it will generally produce another clutch. Indeed, but for the broods hatched from these late clutches, the breed would inevitably have been seriously diminished by the heavy toll taken of their eggs in the spring, and the farmer would thus have lost a most helpful ally; for the plover, feeding as it does exclusively on worms, slugs, and

Nest and Eggs of the Green Plover or Lapwing.

insects, does most valuable service in the fields. The farmers' interest in the survival of the green plover has recently received statutory recognition, and it has been made illegal, in some localities, at any rate, to take plovers' eggs or to expose them for sale after a fixed date about the middle of April. It is practically certain that, in many parts of the country, this year every young plover hatched before April 25 was destroyed by the heavy and unseasonable fall of snow which occurred on that day.

The plover is a very wary bird, and during the breeding season will endeavour, by cunning and apparently frantic manœuvring to draw off the attention of any intruder near its nest. If you wish to locate the nest you must take your bearings not from the bird which flies up at once on your approach and wheels and screams ahead of you almost as far as you choose to follow, but from the other bird, the female, which runs silently for some distance, then takes wing and flies straight away. When the eggs have been only lately laid the plover, for all its anxiety, will not approach you closely, however near you may be standing to the nest, but will attempt by its wild antics to draw you away to its own neighbourhood. When incubation is far advanced, or the young are hatched, the bird is much bolder, and sometimes, if you are standing near the nest or the young, will fly right over you, swooping at times close to your head, making a curious booming sound with its wings not unlike the "drumming" of a snipe.

The young birds can run almost as soon as they are hatched, and are endowed with a self-protective instinct which teaches them at the earliest age to lie motionless on the ground at the approach of danger. In this position their little dark dappled bodies resemble so strongly fragments of soil detached from the molehills or the droppings of sheep that runs over the downs that I have, while actively searching for them, and certain of their immediate neighbourhood, almost trodden upon them unawares. Upon one occasion, when I had successfully surprised a family of plover chicks about two or three days old on a ploughed field, one little fellow, having fallen headforemost between two clods, remained motionless in this uncomfortable

Eggs of the Stone-Curlew.

and ungraceful position during some ten or fifteen minutes while I examined him at leisure—so strong is the instinct which teaches them that the moving object is the first to attract notice.

When once he has dropped, or crouched, at the approach of danger, the young bird will endure a close inspection without attempting to escape, and rather than move will allow himself to be picked up and handled; but when he has been thus disturbed and placed on the ground again, or when he has otherwise become convinced that he is observed, he will begin to run, and will make no further attempt at concealment. When he begins to run another curious trick of instinct reveals itself. The young bird, as he runs, will halt at intervals and bow with his beak on the ground and his tail in the air. Grown plovers, when attempting to lure one away from their nests or young will frequently alight at no great distance and begin to run and bow in the same fashion. Whether this trick has any relation to the pretty stratagem of the broken wing with which so many birds will attempt to lure an intruder away from their young, whether it is intended, for instance, to simulate stumbling exhaustion, or what is its suggestion or intention I have never been able to divine. In the case of a chick which cannot fly, any such trick seems hopelessly inappropriate. I remember another occasion on which I surprised a family of young plovers, rather less than half grown, on a high road. I was bicycling, and at the sight of me they immediately dropped—there were three of them—two in the gutter, and one in the middle of the road. I walked up to this one. He seemed to feel that the case for concealment was hopeless in such surroundings, for he soon got up and ran; but even in that exposed position, and with inviting cover on either side of him, he felt obliged to halt now and again and bow idiotically.

Much more could be written of the ways of the green plover, but perhaps enough has been said to show that he is interesting, not merely as a producer of succulent eggs, but also as a bird of distinctive habits. It is time to refer briefly to another much rarer bird, also known to some as a plover, which returns to its favourite breeding haunts on the downs and in other open places, approximately at the time of year when nesting operations commence among the green plovers. This bird is the stone-curlew, or thicknee, sometimes called the Norfolk plover from the fact that it is comparatively abundant in East Anglia. It is of the same order, but not of the same family as the green plover; it is a larger bird, and bears only a distant resemblance to the plover family. These birds return with great regularity year after year to the same breeding places, and pairs of them may be found every summer on the downs of Dorset, Wilts, and Sussex. They are difficult birds to observe, being extremely wary, running swiftly when first disturbed, then taking wing and flying rapidly right out of sight. The eggs are two in number, blunt at both ends, in sharp contrast to the pointed eggs of the green plover. They are laid on the bare ground generally on stony soil, with no pretence of a nest. The particular pair of eggs illustrated in the accompanying photograph were laid in a field of young oats. The photograph was taken on May 29, 1905, and the young were hatched on June 7—two sturdy chicks, fawn-coloured, with a few darker streaks. I could never find them after that day.

The eggs, like the birds, are very elusive, for owing to their strongly protective colouring, they are most difficult to distinguish from the surrounding soil. This is a matter for congratulation, for the bird is scarce and the eggs much sought after by collectors.

To me the chief attraction of the stone-curlew is its cry, seldom, if ever, heard before sunset. It is a true curlew cry though pitched in a somewhat higher key than that of the common curlew, and, when heard arising out of the silence of the broad downs as one rides homewards in the faint afterglow of the sunset or the deepening twilight, its effect, though weird and somewhat plaintive, is attractively appropriate to the vast solitude and the inexpressible calm that falls upon the downs at night.

## Figures and Tables

**Figure f1:**
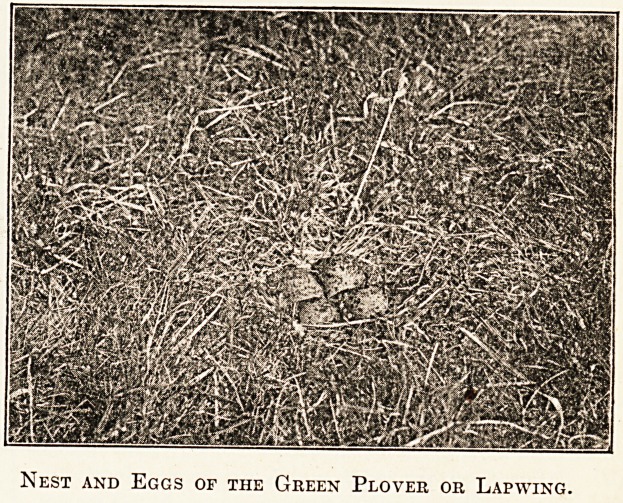


**Figure f2:**